# Protective effect of genistein on radiation-induced intestinal injury in tumor bearing mice

**DOI:** 10.1186/1472-6882-13-103

**Published:** 2013-05-14

**Authors:** Tae Gen Son, Eun Ji Gong, Min Ji Bae, Sung Dae Kim, Kyu Heo, Changjong Moon, Kwangmo Yang, Joong Sun Kim

**Affiliations:** 1 Research Center, Dongnam Institute of Radiological & Medical Sciences (DIRAMS), Jwadong-gil 40, Jangan-eup, Gijang-gun, Busan, Republic of Korea; 2 Department of Veterinary Anatomy, College of Veterinary Medicine and Veterinary Medical Research Center, Chonnam National University, Gwangju, South Korea

## Abstract

**Background:**

Radiation therapy is the most widely used treatment for cancer, but it causes the side effect of mucositis due to intestinal damage. We examined the protective effect of genistein in tumor-bearing mice after abdominal irradiation by evaluation of apoptosis and intestinal morphological changes.

**Methods:**

Mouse colon cancer CT26 cells were subcutaneously injected at the flank of BALB/c mice to generate tumors. The tumor-bearing mice were treated with abdominal radiation at 5 and 10 Gy, and with genistein at 200 mg/kg body weight per day for 1 d before radiation. The changes in intestinal histology were evaluated 12 h and 3.5 d after irradiation. To assess the effect of the combination treatment on the cancer growth, the tumor volume was determined at sacrifice before tumor overgrowth occurred.

**Results:**

Genistein significantly decreased the number of apoptotic nuclei compared with that in the irradiation group 12 h after 5 Gy irradiation. Evaluation of histological changes showed that genistein ameliorated intestinal morphological changes such as decreased crypt survival, villus shortening, and increased length of the basal lamina 3.5 d after 10 Gy irradiation. Moreover, the genistein-treated group exhibited more Ki-67-positive proliferating cells in the jejunum than the irradiated control group, and crypt depths were greater in the genistein-treated group than in the irradiated control group. The mean weight of the CT26 tumors was reduced in the group treated with genistein and radiation compared with the control group.

**Conclusion:**

Genistein had a protective effect on intestinal damage induced by irradiation and delayed tumor growth. These results suggest that genistein is a useful candidate for preventing radiotherapy-induced intestinal damage in cancer patients.

## Background

Radiation therapy plays an important role in the management of cancer. When radiation therapy is directed towards pelvic and abdominal tumors, the malignancy is often successfully controlled or eliminated. The gastrointestinal system often shows clinically relevant lesions induced by physical factors such as ionizing radiation, which is typically used in cancer therapy. Accompanying injury to the surrounding intestinal tissue may result in serious morbidity and occasional mortality. This so-called radiation enterocolitis is a major clinical problem because it is relatively unresponsive to usual therapies and because of the intractable problems it may cause to the patient [1,2]. Cancer patients undergoing radiotherapy have suffered from adverse effects related to the formation of free radicals, which cause oxidative damage to normal cells, including intestinal crypt cells [3]. The protective effects of many compounds against radiation-induced intestinal injuries have been investigated [4-7]. The side effects of irradiation may lead to reduced quality of life and can be dose-limiting, leading to treatment reduction for the patient. Hence, if radioprotective agents are combined with radiotherapy, it may be possible to differentially protect normal cells and kill the cancer cells [4]. The focus of irradiation protection has shifted to investigating the radioprotective potential of natural products, including plants and herbs, in the hope that suitable pharmacological agents, which protect humans against the deleterious effects of ionizing radiation in clinical and other conditions, can be identified [4].

Genistein, a multifunctional soy isoflavone, is a phytochemical that occurs naturally in various plant-derived foods. Genistein scavenges oxygen-derived free radicals and possesses the capacity to activate antioxidant systems; this results in the reduction of free radical lipid peroxidation products and stabilization of cell membrane structure [8-10]. Thus, the antioxidant activity of genistein may protect against radiation-induced cellular damage in cancer patients. Previous studies on the radio-protective effects of genistein in radiation-induced myelosuppressed mice demonstrated that genistein can increase the survival rate of irradiated mice [11-13]. Recently, genistein has been shown to have a radioprotective effect in non-hematopoietic tissues, including the lung and testis [8,14,15]. However, little is known about the protective effect of genistein against radiation-induced intestinal injury.

Genistein also inhibits the growth of cancer cells through the modulation of genes related to the homeostatic control of the cell cycle [16,17]. Understanding the mechanisms involved in the radiosensitization effect of genistein will reveal how it acts as a radiosensitizer in various cancers [15,18-20]. A recent pre-clinical animal study demonstrated enhanced lung tumor eradication and normal lung protection by isoflavones [15]. In this study, we examined the protective effect of genistein on intestinal mucosal damage and the effect of genistein on tumor radiation sensitivity in cancer-bearing mice.

## Methods

### Animals and experimental procedures

Female BALB/c mice (6 weeks old) were purchased from the Central Lab. Animal Inc., (Seoul, Korea) and used after 1 week of quarantine and acclimatization. The animals were maintained in a room at 23°C ± 2°C, with a relative humidity of 50% ± 5%, artificial lighting from 0800–2000 hours, and 13 ~ 18 air changes per hour. They were provided a standard laboratory diet and water *ad libitum*. All experimental procedures were carried out in accordance with the NIH Guidelines for the Care and Use of Laboratory Animals and were approved by the Institutional Animal Care and Use Committee of the Dongnam Institute Radiological and Medical Sciences. The animals were cared for in accordance with the dictates of the National Animal Welfare Law of Korea.

The BALB/c mouse CT26 colon cancer cell line (Korean Cell Line Bank, Seoul, Korea) was cultured in RPMI medium containing 10% fetal calf serum and 1% antibiotics (penicillin, gentamicin and streptomycin; Gibco BRL, Life Technologies Pty. Ltd., Victoria, Australia). CT26 cells (1 × 10^7^ cells per animal in 100 μl PBS) were injected subcutaneously into the flanks of BALB/c mice.

**Figure 1:**
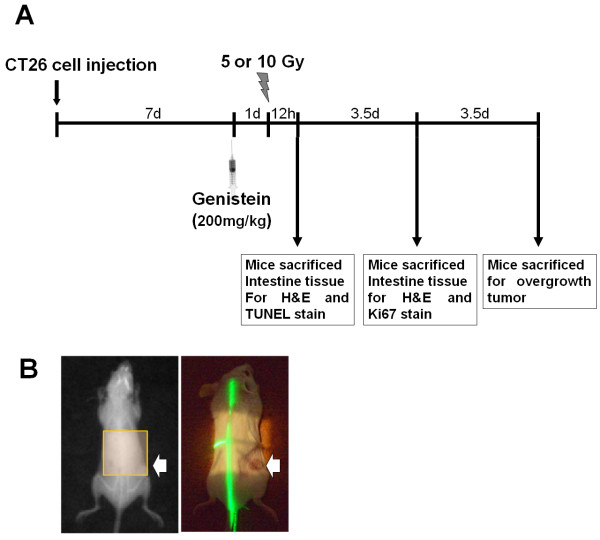
**Reduction of radiation-induced intestinal injury in tumor-bearing mice by genistein: treatment schedule. **(**A**) The mouse CT26 colon cancer cells were injected subcutaneously into the flanks of BALB/c mice. After 7 d of xenograft implantation, when the tumors had reached ~ 5 mm in diameter, the mice were randomly divided into 6 groups and abdominal site exposed to 5 or 10 Gy irradiation in mice. The mice were euthanized 12 h after irradiation, mice tumors were weighed and evaluated the intestinal apoptotic change. For histopathological examination, the mice were euthanized in each group 3.5 day after irradiation, mice tumors were weighed and evaluated intestinal histological change. After 7 d irradiation exposure, mice were euthanized in each group and mice tumors were weighed. (**B**) Abdominal site exposed to irradiation in mice. Arrow is the cancer xenograft lesion.

After 7 d of xenograft implantation, when the tumors had reached ~5 mm in diameter, the mice were randomly divided into 6 groups as follows: (1) vehicle + sham irradiation group (n = 15), (2) genistein + sham irradiation group (n = 15), (3) vehicle + 5 Gy irradiation group (n = 15), (4) genistein + 5 Gy irradiation (n = 15), (5) vehicle + 10 Gy irradiation (n = 15), and (6) genistein + 10 Gy irradiation (n = 15), according to the treatment schedule (Figure 1A). The five mice were euthanized randomly in each group 12 h after irradiation, mice tumors were weighed and evaluated the intestinal apoptotic change. For histopathological examination, the five mice were euthanized in each group 3.5 d after irradiation, mice tumors were weighed and evaluated intestine histological change. After 7 d after irradiation, mice were euthanized in each group and mice tumors were weighed.

Genistein and polyethylene glycol of molecular weight 400 (PEG) were obtained from Sigma Chemical Company (St Louis, MO). Genistein was dissolved in PEG on the day of the experiment by sonication for 20 sec (Heat Systems-Ultrasonics Inc., Plainview, NY, USA). Genistein was administered into the dorsal subcutaneous space at a dose of 200 mg/kg body weight at 24 h prior to irradiation; the dose and dosage were considered optimum for radioprotection, as reported previously [11,13] (Figure 1A).

The anesthetized mice were positioned on a tray, and the intestine and cancer lesion were confirmed to be in the radiation field. The mice were imaged by *in vivo* X-ray micro-computed tomography (NFR Polaris-G90, Nanofocusray, Jeonju, Korea) before radiation exposure as the pretreatment control. The mice received abdominal body irradiation at 5 and 10 Gy doses using 6 MV high-energy photon rays (ELEKTA, Stockholm, Sweden) at a dose rate of 3.8 Gy/min for the evaluation of intestinal injury and cancer changes. Sham-irradiated mice were treated in exactly the same manner as the irradiated mice but without irradiation (Figure 1B).

### Apoptosis assay

The mice (5 mice in each group) were euthanized 12 h after irradiation (5 and 10 Gy), based on a report showing that the maximum frequency of apoptotic cells was observed after irradiation [21]. Small intestines were fixed in 10% buffered formalin and embedded in paraplast wax. Sections (4-μm thick) were cut and stained using the TdT-mediated dUTP-biotin nick end labeling (TUNEL) technique with a commercial kit (ApopTag Plus Peroxidase *In Situ* Apoptosis Detection kit, Intergen Co., Burlington, MA, USA). Apoptotic cells were counted in the longitudinal crypt sections showing a large portion of cells in the crypt base and lumen and at least 17 cells along the crypt column using an optical microscope. The cells were recorded as single cells based on their size and clustering when several apoptotic fragments were believed to represent the remains of a single cell. Forty crypt sections were recorded for each mouse [5,6].

### Jejunal crypt assay and morphological changes

The mice (5 mice in each group) were euthanized 3.5 d after gamma irradiation (5 and 10 Gy). The small intestines were fixed in 10% buffered formalin and embedded in paraplast wax to prepare 4-μm thick tissue sections of jejunum for hematoxylin-eosin staining. Two sections from 4 different parts of the jejunum from each animal were prepared for histological examination. The regenerating crypts and villi in the jejunal cross-section were then counted. To analyze morphological changes, all samples were sectioned and reoriented in successive slices to identify those containing the longest villi. This technique was used because it yielded more homogenous results than those of standard techniques based merely on the measurement of the 10 longest villi in a single slice per sample. The length of the 10 longest villi, crypts, and basal lamina of 10 enterocytes in each sample were measured. Ten measurements were obtained per animal, for a total of 50 measurements per group [5,7]. Images of intestinal sections were obtained using a digital camera mounted on a microscope (Leica DM IRBE, Leica Micro Systems GmbH, Wetzlar, Germany). Quantification was performed using image analysis software (Leica QWin, Leica Microsystems, Wetzlar, Germany).

To assess cell proliferation in tissue samples, the proliferation antigen Ki-67 was analyzed by immunocytochemistry using a polyclonal rabbit anti-Ki-67 antibody (Acris Antibodies GmbH, Hiddenhausen, Germany; diluted 1:500). Bound antibodies were detected with avidin-biotin peroxidase (Elite kit, Vector, Burlingame, CA, USA), and the peroxidase reaction was developed using a diaminobenzidine substrate kit (Vector). As controls, the primary antibody was omitted from a few test sections in each experiment. The sections were counterstained with hematoxylin before being mounted.

### Effect of genistein in combination with radiotherapy on colon cancer in tumor-bearing mice

Tumor overgrowth occurred 14 d after injection of cancer cells (7 d after irradiation). To monitor tumor size of mice, tumors were excised and weighed at different time points 12 h, 3.5 d, and 7 d after irradiation exposure.

### Statistical analysis

The data are reported as the means ± SEM. The data were analyzed by one-way analysis of variance (ANOVA) followed by a Student–Newman–Keuls *post hoc* test for multiple comparisons. In all cases, a *P* value of <0.05 was considered significant.

## Results

### Anti-apoptotic effect of genistein in the jejunal crypts

**Figure 2:**
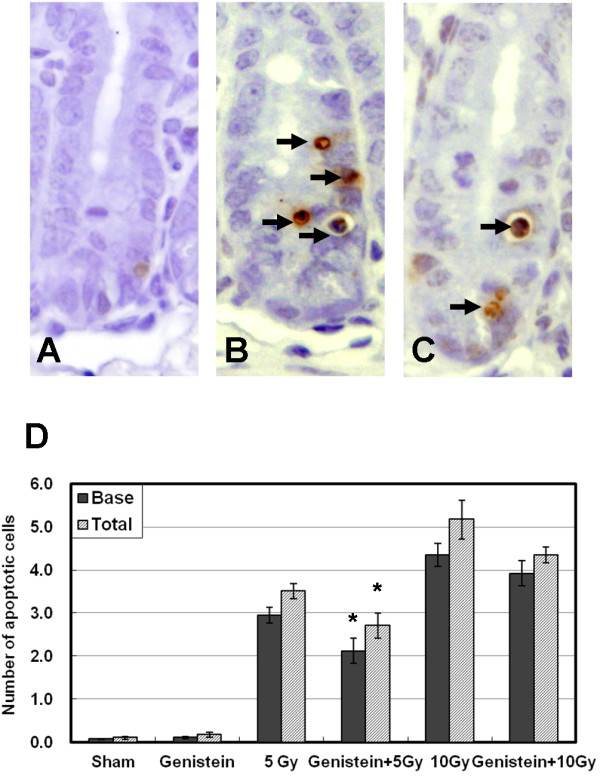
**Photomicrographs of the apoptotic changes in the jejunal crypts revealed by TUNEL assay 12 h after 5 Gy gamma irradiation. **(**A**) Sham control, (**B**) irradiation control, and (**C**) genistein treatment prior to irradiation. (**A**) A small number of crypt cells exhibited apoptosis in the sham control mice. (**B**) Irradiation increased the expression of apoptotic nuclei in the crypt cells of the jejunum. (**C**) The number of apoptotic cells decreased slightly in the group treated with genistein compared with the irradiation control group (**A**, **B**, and **C**). (**D**) Bar graphs showing the number of apoptotic cells per crypt in the jejunum sections, as determined by the TUNEL method, in the sham control group, irradiation control group, and genistein-pretreated group exposed to 5 Gy irradiation. The data are shown as the means ± SEM for 5 mice in each group. *P < 0.05 as compared with the irradiation control group.

The number of apoptotic cells in the jejunal crypts was determined by the TUNEL method. Apoptosis was easily recognized by TUNEL staining of entire apoptotic bodies. Most of the apoptotic cells were observed in the putative stem cell zone located at the bottom (base) of the jejunal crypts (Figure 2). Only a small number of crypt cells exhibited apoptosis in the sham-irradiated mice (Figure 2A and D). Irradiation increased the expression of apoptotic nuclei in the jejunal crypts in a dose-dependent manner (5 and 10 Gy), as demonstrated by the TUNEL method (Figure 2B and D). The number of apoptotic cells decreased significantly in the genistein-treated group compared with the vehicle-treated 5 Gy irradiated group (Figure 2C and D) (*P* < 0.05 vs. irradiation group at 12 hours after 5 Gy). Although the administration of genistein decreased the average number of apoptotic cells in the crypts in 10 Gy irradiated group, there was no significant difference between the vehicle- and genistein-treated groups exposed to 10 Gy irradiation (Figure 2D).

### Protective effect of genistein on intestinal morphological changes

**Figure 3:**
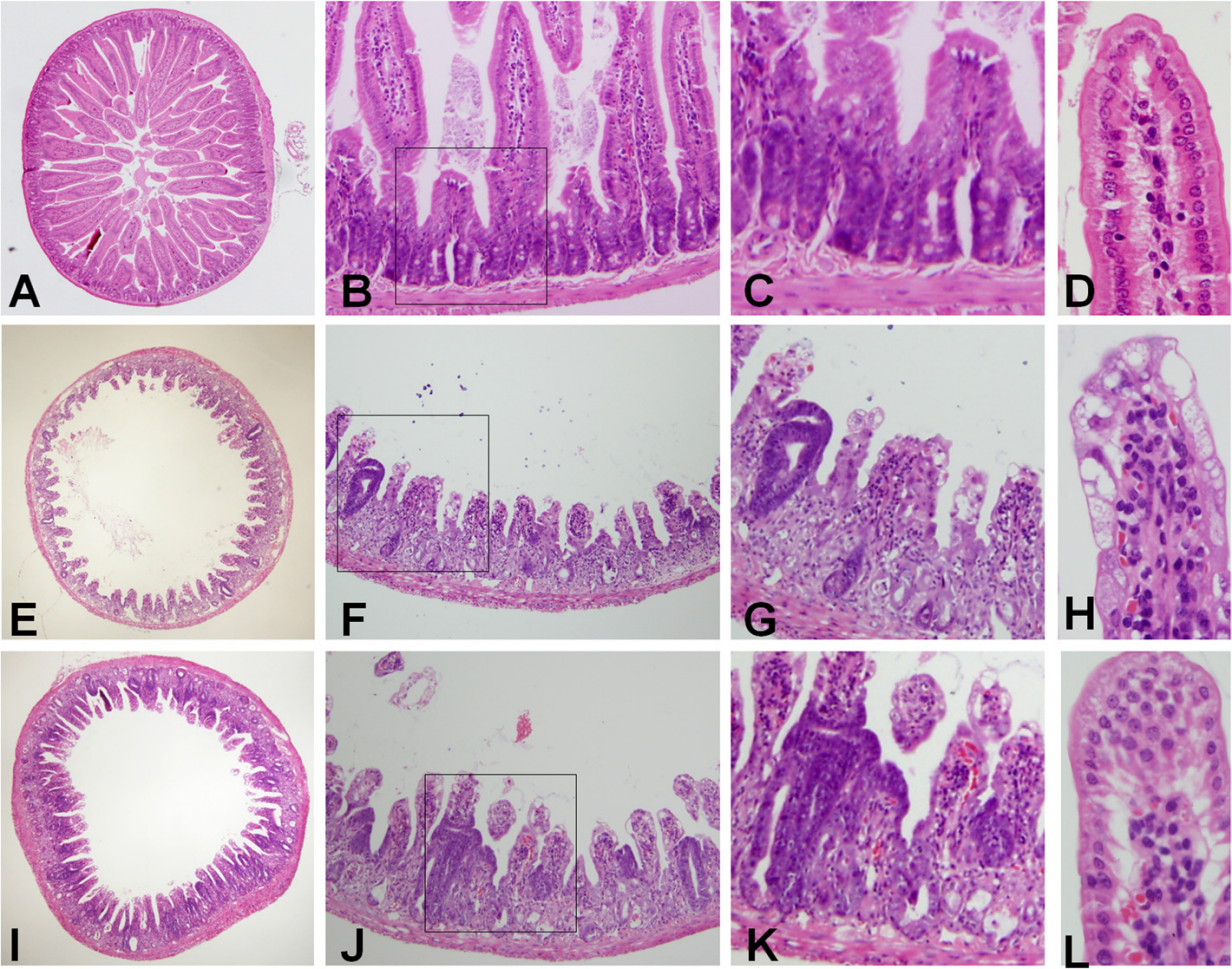
**Photomicrographs showing crypt survival (A, E, and I), villus height (B, F, and J), crypt depth (C, G, and K), and lengths of the basal lamina of 10 enterocytes (D, H, and L) in the jejunal circumference, as revealed by hematoxylin and eosin staining.** (**A**–**D**) Sham control group, (**E**–**H**) irradiation control group, and (**I**–**L**) genistein-pretreated group 3.5 d after 10 Gy irradiation. Intestinal sections from the sham group show normal morphology. The genistein-pretreated group exposed to 10 Gy irradiation exhibited increased crypt survival, villi length, and crypt depth when compared with the irradiation control group exposed to 10 Gy irradiation at this magnification and in cross section. The genistein-pretreated group (**I**) exhibited a shorter basal lamina length when compared with the 10 Gy irradiation control group (**H**). (**A**, **E**, and **I**) magnification × 40; (**B**, **F**, and **J**) magnification × 100; (**C**, **G**, and **K**) magnification × 200; (**D**, **H** and **L**) magnification × 400.

Table 1 summarizes the morphological changes observed in jejunal mucosa 3.5 d after 10 Gy irradiation. The morphological changes observed in mouse jejunum are shown in Figure 3. No significant changes in intestinal morphology were seen in the 5 Gy irradiated mice as compared with the sham group (data not shown). Tissue sections of intestine from the sham group and the genistein-treated non-irradiated group showed normal morphology (Figure 3A–D, Table 1).

**Table 1:** Effect of genistein treatment on histologic changes in the mouse small intestine 3.5 d after 10 Gy irradiation

	**Sham**	**Genistein**	**IR (10 Gy)**	**Genistein + IR (10 Gy)**
Number of crypts/circumstance	120.7 ± 2.5	109.1 ± 8.2	14.6 ± 2.5	24.6 ± 2.6*
Number of villi/circumstance	40.6 ± 2.7	41.1 ± 3.8	32.6 ± 1.8	35.5 ± 1.7
Villus height (μm)	398.6 ± 13.8	378.4 ± 18.1	124.0 ± 7.2	186.2 ± 12.5**
Crypt depth (μm)	89.6 ± 5.8	90.1 ± 4.8	98.9 ± 4.2	136.8 ± 7.1**
Basal lamina length (μm/10 enterocytes)	44.6 ± 3.0	41.4 ± 6.2	123.1 ± 10.4	95.7 ± 5.9*

Data are shown as means ± SEM (*P < 0.05, **P < 0.01 vs. IR control group).

The number of surviving crypts in the irradiation group decreased significantly 3.5 d after 10 Gy irradiation (Figure 3E and F, Table 1). The number of surviving crypts increased significantly in the genistein-treated group (Figure 3I and J, Table 1) compared with the irradiation group (*P* < 0.01 vs. irradiation group 3.5 d after 10 Gy irradiation). These results indicate that genistein pretreatment significantly improved the survival of jejunal crypts in irradiated mice.

The height and number of villi decreased significantly in mice 3.5 d after 10 Gy irradiation compared with the sham control group, demonstrating the injurious effects of irradiation on the jejunal mucosa (Figure 3E and F). The villus height was higher in the genistein-pretreated groups (Figure 3J) than in the irradiation group with vehicle treatment (Table 1; *P* < 0.05 vs. irradiation group 3.5 d after 10 Gy irradiation). Although a higher number of villi was observed in the genistein-treated group (Figure 3I, Table 1) than in the irradiation control group 3.5 d after 10 Gy irradiation, there was no significant difference.

The crypt depths were greater in the mice that received irradiation, reflecting the intestinal response that follows injury and leads to repair (Figure 3G, Table 1). However, the crypt depths were increased in the genistein-treated group compared with the irradiation group (Figure 3K, Table 1; *P* < 0.05 vs. irradiation group 3.5 d after 10 Gy).

Comparison of the lengths of basal lamina per 10 enterocytes in the mouse jejunum is shown in Figure 3D, H, and L. The lengths of the basal lamina of enterocytes were increased in the irradiation groups compared to the control group. However, the genistein-pretreated group exhibited shorter enterocyte basal lamina than that of the irradiation group, indicative of reduced injury in the genistein-pretreated group (Table 1; *P* < 0.05 vs. irradiation group 3.5 d after 10 Gy).

**Figure 4:**
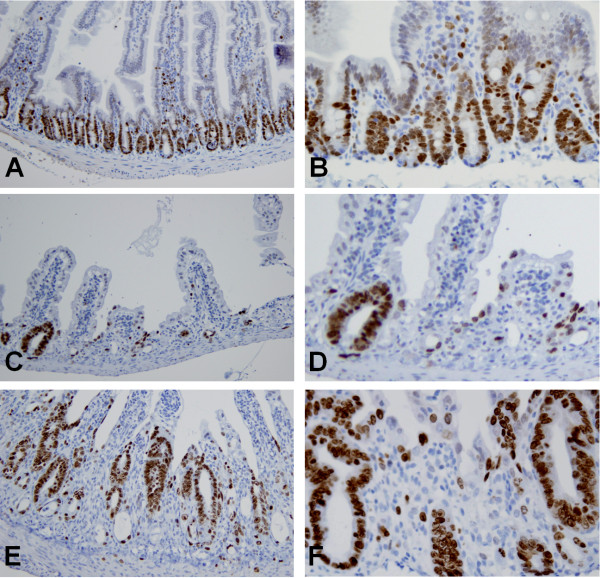
**Photomicrographs of progenitor cells in the jejunum circumference stained with an antibody for Ki-67. **(**A** and **B**) Sham control group, (**C** and **D**) irradiation control group, and (**E **and **F**) genistein-pretreatment group 3.5 d after 10 Gy irradiation. Most of the Ki-67-positive cells were detected in the jejunal crypts. The number of Ki-67-positive cells decreased in irradiated mice, which corresponded to surviving crypts at 3.5 d after 10 Gy irradiation. However, higher Ki-67 expression was observed in crypts of increased depth in irradiated groups compared with the sham control group. A greater number of Ki-67-positive cells was observed in the genistein-treated group than in the irradiation control group at this magnification. (**A**, **C**, and **E**) magnification × 100. (**B**, **D**, and **F**) magnification × 400.

Proliferating cells were identified by immunohistochemical staining of Ki-67 [7,22] (Figure 4). Most Ki-67-positive cells were detected in the jejunal crypts. The number of Ki-67-positive cells decreased in irradiated mice, corresponding to the decrease in surviving crypts 3.5 d after 10 Gy irradiation. A greater number of Ki-67-positive cells was observed in the genistein-pretreated group compared with the irradiation group, similar to the increase in crypt survival and depth (Figure 4). The difference in Ki-67 expression between the irradiation group and the genistein-pretreated group indicates the better survival of crypts in the genistein-pretreated group.

### Effect of genistein on sensitivity to radiation in CT26 cell xenografted mice

After 12 hr, 3.5 and 7 d, mice (5 mice in each group) with tumors were euthanized, and the tumors were weighed. Although genistein alone and 5 Gy irradiation alone decreased the size of the tumors compared with the sham group, the difference was not significant. Compared with controls, 10 Gy irradiation alone and combination therapy resulted in a significant decrease in tumor size after 7 d (*P* < 0.05 vs. sham group 7 d after 10 Gy). Combined treatment (10 Gy + genistein) produced the best tumor regression and growth inhibition among the 6 groups, with no significant difference between the vehicle and genistein treated groups (Table 2; P = 0.5, 10 Gy group vs. 10 Gy + genistein group).

**Table 2:** Effect of genistein treatment on tumor weight in CT26 cell xenografted BALB/c mice 12 h, 3.5 and 7 day after 5 and 10 Gy irradiation

	**Sham**	**Genistein**	**IR (5 Gy)**	**Genistein + IR (5 Gy)**	**IR (10 Gy)**	**Genistein + IR (10 Gy)**
Post IR 12 h	0.22 ± 0.05	0.2 ± 0.03	0.24 ± 0.04	0.17 ± 0.04	0.18 ± 0.03	0.18 ± 0.04
Post IR 3.5 d	0.45 ± 0.08	0.42 ± 0.07	0.45 ± 0.09	0.44 ± 0.05	0.41 ± 0.03	0.39 ± 0.04
Post IR 7 d	0.75 ± 0.06	0.66 ± 0.04	0.72 ± 0.06	0.63 ± 0.06	0.58 ± 0.04*	0.53 ± 0.06*

The data are shown as means ± SEM for 5 mice in each group. (,*P < 0.05, vs. sham group).

## Discussion

This study examined the levels of radiation-induced damage to small intestinal cells to determine the protective effect of genistein. The results showed that genistein has the ability to regulate progenitor cell survival and cell death in the small intestine.

To evaluate radiation-induced intestinal damage, we determined the number of villi and crypts, villus height, crypt depth, and length of the basal lamina of enterocytes after irradiation. The height of the villi and number of surviving crypts may be very sensitive and suitable biodosimetric markers after irradiation [6,7,23]. Cell proliferation in the crypts retards the developing intestinal damage, but after higher doses of radiation, the recovery mechanism of cell repopulation is insufficient [24]. The acute effects of irradiation on the intestinal mucosa are generally ascribed to inhibition of mitosis in the crypts [25]. This loss of proliferative function results in the development of epithelium and renders the intestine permeable to luminal bacteria and antigens, which may exacerbate mucosal inflammation and dysfunction or cause bacteremia [2]. The expression of Ki-67, a proliferative marker in the jejunum, increased in the genistein-treated group; this may indicate the recovery of intestinal damage after irradiation. Thus, genistein can attenuate various morphological changes due to intestinal injury after irradiation. These results demonstrate that genistein provides significant protection against intestinal injury following irradiation. Although clinical radiotherapy usually involves fractioned radiation doses and not a single therapy, this study aimed to identify the radioprotective effect of genistein on intestinal injury induced by radiation rather than its anti-cancer effect. Therefore we used a single dose of radiation to induce intestinal injury because it is difficult to induce intestinal injury by fractioned radiation. Compared with fractioned radiation, the single exposure resulted in considerably less intestinal injury, indicating a substantial recovery during the 6 hr span between radiation fractions [26].

Most irradiation damage in biological systems is caused by the generation of reactive oxygen species. Various mechanisms, including the prevention of damage through inhibition of free radical generation or their intensified scavenging, enhancement of DNA and membrane repair, replenishment of dead hematopoietic and other cells, and stimulation of immune cell activity, are considered important for radioprotection [4]. The antioxidative properties of genistein, combined with its capacity to activate antioxidant systems, reduce lipid peroxidation and stabilize cell membrane structure [9,10]. Arora et al. found that soy isoflavones can hinder the diffusion of free radicals and thereby decrease the kinetics of free radical reactions, which might help to stabilize cell membrane structure [27]. In our previous study, we showed that genistein can significantly decrease the level of radiation-induced reactive oxygen species in the testis, suggesting that genistein protects against radiation-induced testicular injury via a protective mechanism that includes antioxidative activity [8]. Thus, the antioxidant activity of genistein, i.e., its ability to protect against irradiation-induced cytogenetic damage, may contribute to its radioprotective action.

Moreover, genistein binds to the estrogen receptor due to its biphenolic structure and may mimic or modulate the actions of endogenous estrogens [28]. These include its estrogenic activity and its role in signal transduction pathways where it is an inhibitor of topoisomerase, protein kinases, and caspases involved in apoptotic pathways [16,29]. Anti-apoptotic effects have been reported to be associated with protection after radiation-induced intestinal injury [5-7]. Furthermore, estrogen has been shown to protect the gut in a model of inflammatory bowel disease, and this effect is mediated via the estrogen receptor [30]. Another study indicated that estrogen also attenuates intestinal injury following trauma-hemorrhage via upregulation of the Akt pathway [31]. Although further studies are required to better understand the protective effects of genistein, estrogen may contribute to the anti-inflammatory effect of genistein along with anti-apoptotic pathways and the Akt pathway.

Genistein administration stimulated serum granulocyte-colony stimulating factor (G-CSF) after sham irradiation or gamma-irradiation [32]. Recent study showed the radioprotective effect of some agents is mediated the through G-CSF induction [33,34]. Further previous our study also showed that G-CSF attenuated intestinal damage after radiation exposure [7]. G-CSF activates several signaling pathways to promote survival and proliferation, and it also protects against apoptosis after irradiation exposure [7,35,36]. The induction of G-CSF by genistein is also responsible for protection against radiation injury.

Furthermore, genistein has gained increasing attention because of its association with beneficial effects in cancer chemoprevention [15,18,19,37,38]. This study showed that a single treatment of genistein alone does not decrease tumors compared with the sham group, no significant difference was observed between treatment with vehicle and genistein with radiation exposure. Although, the single treatment of genistein has not effects on the cancer cell in this study, other studies have demonstrated that continuously treatment of genistein shows additive benefits in tumor radiotherapy, resulting in greater therapeutic efficacy [18,19]. This study showed that combination therapy with 10 Gy irradiation and genistein produced the best tumor regression and growth inhibition among the groups. The results showed that combination therapy with 10 Gy irradiation and genistein produced the best tumor regression and growth inhibition among the groups. Some reports have shown that genistein used alone *in vivo* as well as *in vitro* can delay the growth of tumors [37,38]. Some authors indicated that genistein in combination with other agents can delay tumor growth by inhibiting angiogenesis [39]. Hillman et al. [18,19] also showed that genistein combined with irradiation for prostate cancer and renal cancer led to improved control of primary tumor growth and metastasis to lymph nodes compared with genistein or irradiation alone [18-20]. Further, a recent study showed that soy isoflavones augment the destruction of lung cancer by radiation and also mitigate vascular damage, inflammation, and lung fibrosis caused by radiation injury to normal lung tissue [15].

Although the cancer patients might benefit from radiotherapy, it is not devoid of side effects. Among patients receiving abdominal radiotherpay more than 70% develop gastro intestinal symptoms during treatment [40]. Our findings show that genistein reduces various parameters related to intestinal injury caused by radiation exposure, but has not an effect on the tumor growth in cancer-bearing mice. Therefore, genistein may be clinically useful for colon cancer patients who require radiotherapy.

## Conclusion

Genistein has a potential role in protection against radiation-induced intestinal damage while providing radiosensitizing effect on tumor cells. Genistein treatment protected mice from apoptosis in the jejunal crypt 12 h after 5 Gy irradiation and restored various parameters related to intestinal injury 3.5 d following 10 Gy irradiation in tumor-bearing mice. The weight of cancers was lesser in genistein- and irradiation-treated mice than in mice receiving each single treatment. In conclusion, genistein is a candidate drug that may protect intestinal cells from the side effects of radiation therapy and provide a radiosensitizing effect on tumor cells.

## Abbreviations

PEG: Polyethylene glycol of molecular weight 400; TUNEL: TdT-mediated dUTP-biotin nick end labeling; G-CSF: Granulocyte-colony stimulating factor.

## Competing interests

The authors declare that they have no competing interests.

## Authors’ contributions

TS, EJG, MB, SDK, KH, and JSK participated in the design of the study, data analyses, and manuscript preparation. KY created the partial body irradiation model. CM participated in the intestinal histoloigical analysis. All authors read and approved the final manuscript.

## Pre-publication history

The pre-publication history for this paper can be accessed here:

http://www.biomedcentral.com/1472-6882/13/103/prepub
